# COVID-19 infection could be a risk factor for dementia?

**DOI:** 10.1192/j.eurpsy.2022.1323

**Published:** 2022-09-01

**Authors:** P. Costa, I. Pinto, P. Branco

**Affiliations:** 1 Centro Hospitalar Psiquiátrico de Lisboa, Clínica 2 - Psicogeriatria, Lisboa, Portugal; 2 Centro Hospitalar Psiquiátrico de Lisboa, Clínica 1 - Unidade Partilhada, Lisboa, Portugal

**Keywords:** “COVID-19”, “SARS-CoV-2”, “pandemics”, “cognitive impairement”, “dementia”, “risk factor”

## Abstract

**Introduction:**

Since the COVID-19 pandemic start in early 2020, there have been reports of a high prevalence of neuropsychiatric symptoms. Cognitive impairment is being increasingly recognized as an acute and possibly long-term sequel of the disease. According to recent data, limited evidence point to SARS-CoV-2 having a preferential neurotropism for the frontal lobes, as suggested by behavioral and dysexecutive symptoms, frontotemporal hypoperfusion on MRI, EEG slowing in frontal regions, and frontal hypometabolism on 18F-FDG-PET. Nevertheless, there isn’t a specific biomarker.

**Objectives:**

Brief literature review about the relationship between COVID-19, cognitive impairement onset and risk for dementia.

**Methods:**

Non-systematic review through PubMed research using the terms “COVID-19”, “SARS-CoV-2”, “pandemics”, “cognitive impairement”, “dementia” and “risk factor”.

**Results:**

Direct neuronal infection via angiotensin-converting enzyme 2 receptor (ACE2R), hyperinflammation, brain ischemia related to respiratory failure or thromboembolic strokes, and severe psychological stress are the mechanisms more associated with a deleterious effect on cognition. The relation between SARS-CoV-2 infection and neurodegenerative diseases is still unclear. However, the high expression of the ACE2R in the brain, may explain the acute brain damage and could also be the basis for later neurodegenerative changes. The potentially long-term nature of the deficits makes it important to do an early identification, management, rehabilitation and follow-up of the patients exhibiting cognitive symptoms.

**Conclusions:**

Given the reports of brain damage by SARS-CoV-2, there are concerns that this damage may substantially increase the incidence of neurodegenerative diseases and promote dementia. Further long-term studies may be required to identify the relationships between SARS-CoV-2 infection and risk for dementia.

**Disclosure:**

No significant relationships.

